# Extensive Unpredictable Pancreas Cancer Inter-fraction Motion: A Case Report

**DOI:** 10.7759/cureus.5047

**Published:** 2019-06-30

**Authors:** Sangjune L Lee, Michael Velec, Pablo Munoz-Schuffenegger, Teo Stanescu, Laura Dawson

**Affiliations:** 1 Radiation Oncology, University of Toronto, Toronto, CAN; 2 Radiation Medicine, Princess Margaret Hospital/University Health Network, Toronto, CAN; 3 Hematology – Oncology, Pontifical Catholic University of Chile, Santiago, CHL; 4 Medical Physics, Princess Margaret Cancer Centre, Toronto, CAN; 5 Radiation Oncology, Princess Margaret Cancer Centre, Toronto, CAN

**Keywords:** pancreatic cancer, conformal radiation, four-field box, organ motion

## Abstract

We present a case of locally advanced pancreatic cancer with duodenal invasion treated with consolidative chemoradiation, where extensive unpredictable interfraction motion was observed. Initially, two attempts were made to treat with volumetric modulated arc therapy technique. However, due to substantial interfractional motion of the pancreatic head mass relative to the regional nodal areas, the patient was eventually replanned and treated with a four-field box technique. This case highlights the difficulty in delivering conformal radiation to the pancreas and quantifies the movement of the target, the adjacent biliary stent, and regional nodes.

## Introduction

Patients with pancreatic cancer have a five-year overall survival of 6%, and only 15% of presenting cases are resectable [1]. Effective and safe radiation therapy delivery to the pancreas is challenging due to the proximity of numerous moving, dose-limiting organs at risk [2]. We present a case of locally advanced pancreatic cancer treated with consolidative chemoradiation, where extensive unpredictable inter-fraction motion was observed.

## Case presentation

A 75-year-old woman with a gastrointestinal stromal tumor resected eight years prior presented with a 44-mm mass in the head of the pancreas encasing the superior mesenteric artery (SMA), a 13-mm node at the proximal SMA, invasion of the duodenum, and biliary obstruction on CT. An endoscopic retrograde cholangiopancreatography (ERCP), metal stent insertion and brushings confirmed an adenocarcinoma. She then received nine cycles of fluorouracil, leucovorin, irinotecan and oxaliplatin (FOLFIRINOX) over eight months. After seven months of chemotherapy, CT showed an excellent response with a residual 30 mm hypoattenuating mass. Consolidative concurrent chemoradiation, 52.5 Gy in 30 daily fractions, with biweekly low dose gemcitabine was offered [3].

**Figure 1:**
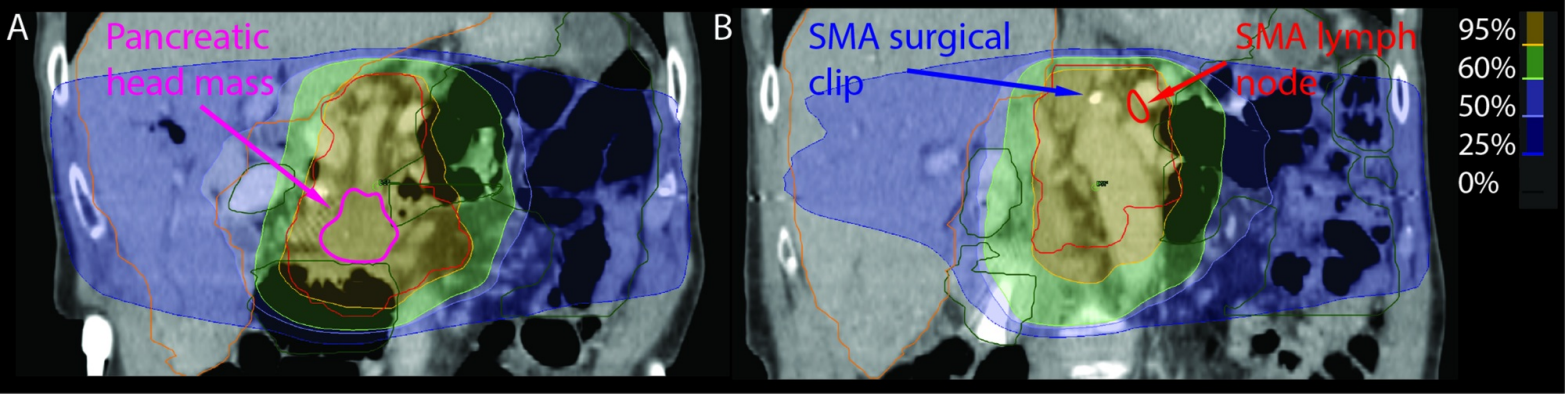
Coronal views of the isodose distribution for the original VMAT plan. The pancreatic head mass (A) is located anteriorly and inferiorly to the SMA node (B). A metal clip from a previous surgery, superior to the take-off of the SMA, served as a fiducial for the SMA node on CBCT. SMA: Superior mesenteric artery; VMAT: Volumetric modulated arc therapy; CBCT: Cone-beam computed tomography.

**Figure 2:**
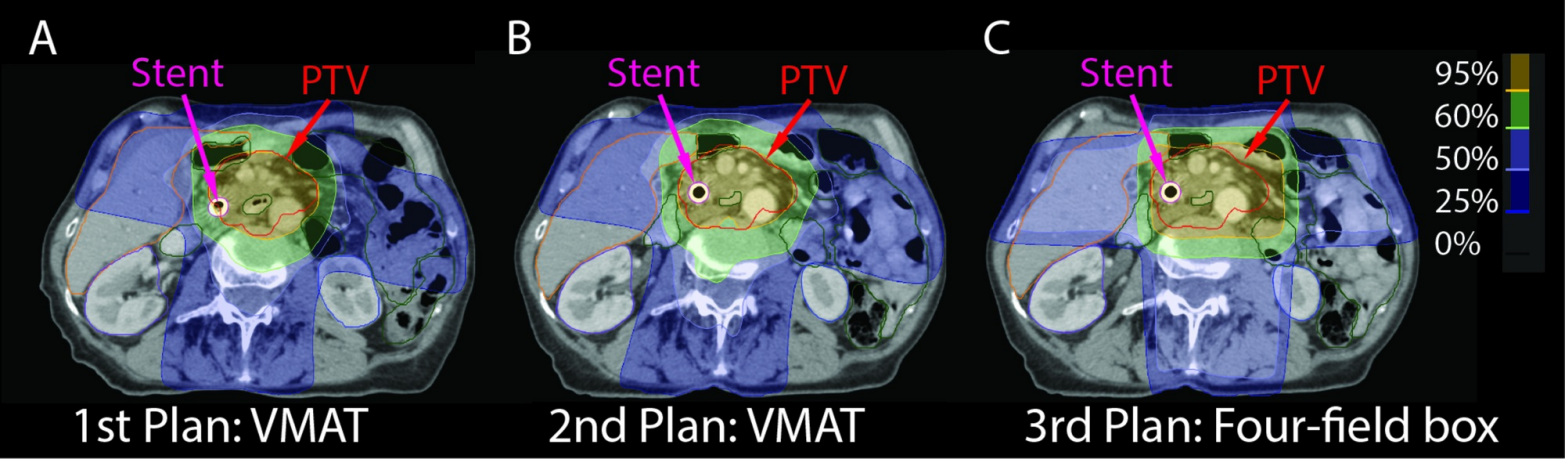
Axial views of the isodose distribution for each of the radiation plans. First plan: VMAT (A), second plan: VMAT (B), third plan: FFB (C). Plans were scaled to the original prescription of 52.5 Gy in 30 fractions for comparison. FFB: Four-field box; VMAT: Volumetric modulated arc therapy; PTV: Planning target volume.

At simulation, a helical exhale breath-hold CT with intravenous and oral contrast and a 4DCT were obtained. A 10-mm gross target volume (GTV) expansion plus initially involved nodes were combined with regional nodal regions to create clinical target volumes (CTVs) on helical, inhale and exhale CT images [4]. The combined CTVs formed an internal target volume (ITV) that was isotropically expanded by 5 mm to create the planning target volume (PTV). The first volumetric modulated arc therapy (VMAT) plan (Pinnacle v9.6, Philips Medical Systems, Madison, WI) was created and delivered in free breathing (Figure 1 and Figure 2).

**Figure 3:**
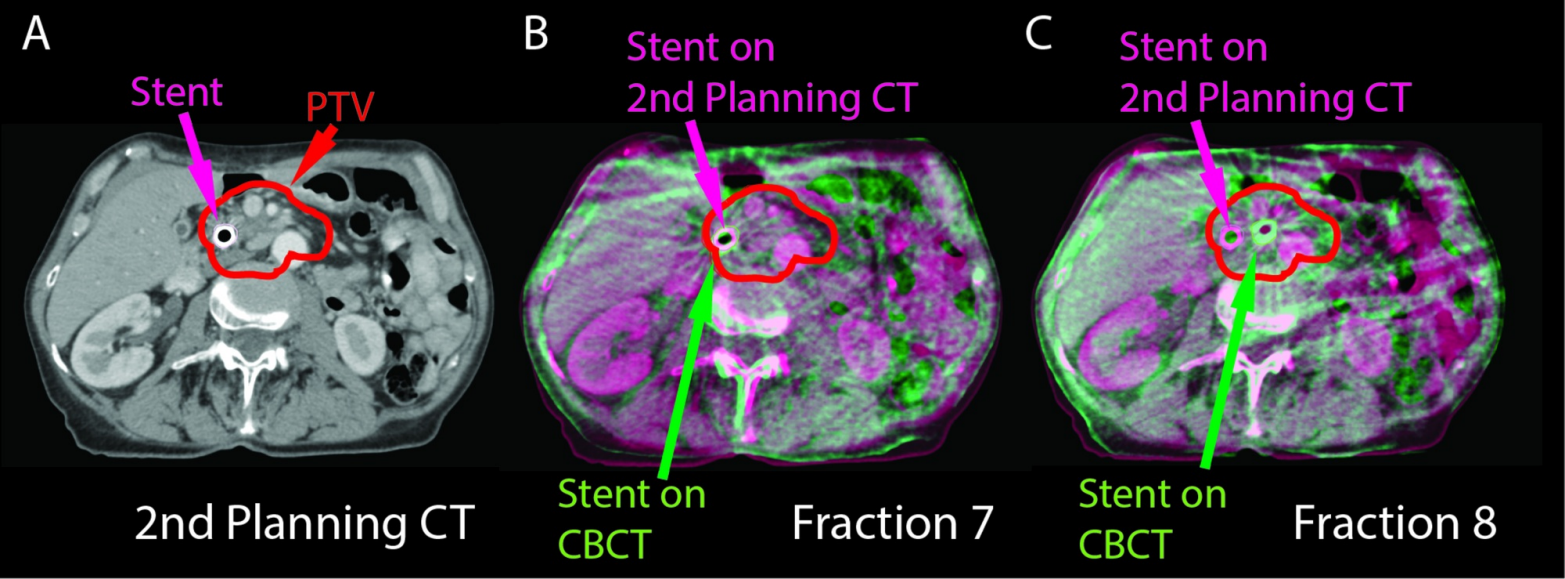
CT and CBCT fusions. Second planning CT (A) and fused planning CT with CBCTs are shown (B-C). The planning CT is in purple and the CBCT is in green. Although the stents were aligned on fraction 7 (B), on fraction 8 the stent moved 20 mm medially with maintained vertebral body alignment (C). PTV: Planning target volume; CBCT: Cone-beam computed tomography.

Daily image-guidance was based on cone-beam CT (CBCT) with a bone match, followed by a review of the stent position as it was adherent to the pancreatic head. CBCT showed wide stent variations in position and fluctuations in the volume of bowel gas despite the patient’s adherence to our dietary preparation instructions of no food for two hours prior to treatment. A repeat CT simulation for replanning on fraction 4 was ordered to account for the shift in the stent and pancreas, relative to the stable SMA. The GTV could be covered with the original plan by shifting the patient position laterally; however, this shift led to under-coverage of the SMA nodal CTV. A surgical clip from eight years prior, superior to the SMA root and less than 20 mm right of the suspicious SMA node, was identified as a surrogate for the SMA node on CBCT. A second VMAT plan, with a 6-mm isotropic ITV to PTV expansion, was initiated on fraction 6. However, the changes in stent position by up to 30 mm continued to be observed (Figure 3).

To investigate possible intra-fractional motion, the patient underwent MRI on fraction 14 with 2D-cine acquisition through the target, an 8-mm slice thickness, in-plane resolution of 1 mm and a temporal resolution of 3 Hz. Pancreas’ respiratory motion was approximately 10 mm superior-inferior, 8 mm lateral, and 5 mm anterior-posterior. Inhale breath-hold MR images taken 30 minutes apart showed less than 10 mm stent motion. Overall, intra-fractional motion was less than inter-fractional motion. Due to concern of the compromised nodal CTV, the patient was replanned once again with a ‘robust’ four-field box (FFB) technique using the second planning CT starting on fraction 17. On this third plan, the ITV to PTV expansions were 7 mm anterior-posterior, 7 mm right, 17 mm left, and 7 mm superior-inferior. These expansions were specified to cover the inter-fraction motion seen on previous fractions.

**Figure 4:**
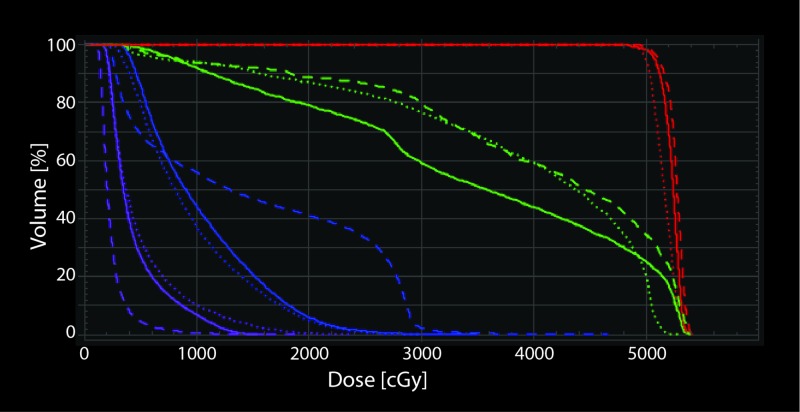
Dose-volume histogram for the three treatment plans used to treat this patient. This assumes all 30 fractions were delivered with each plan. First plan with VMAT (solid line), second plan with VMAT (dotted line), and third plan with FFB (dashed line) are shown with prescriptions set to 52.5 Gy in 30 fractions for comparison. PTV (red), duodenum (green), left kidney (blue) and right kidney (purple) are shown. FFB plan has higher doses to the organs at risk, except for the right kidney. PTV: Planning target volume; CBCT: Cone-beam computed tomography; FFB: Four-field box; VMAT: Volumetric modulated arc therapy.

**Figure 5:**
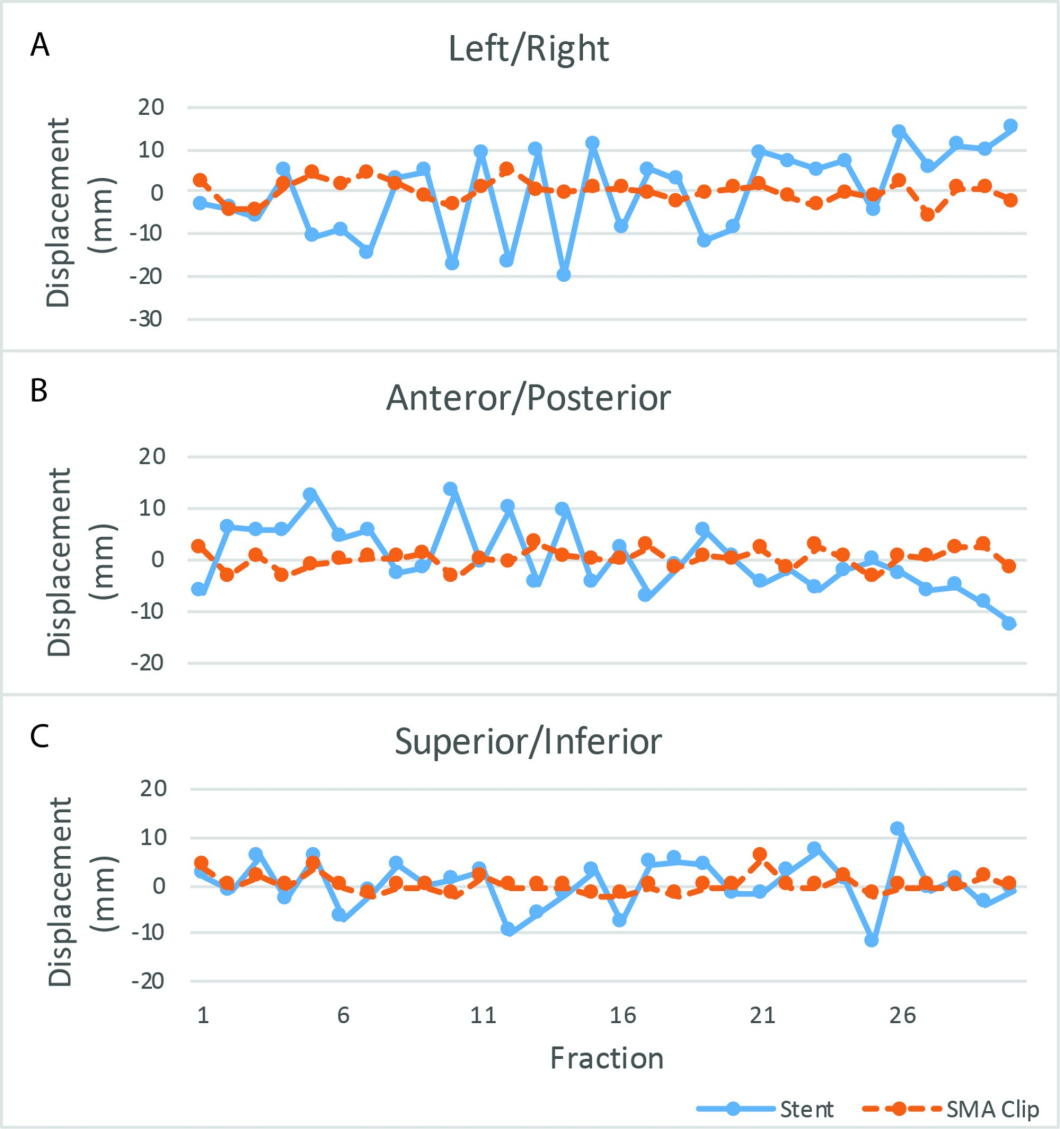
Stent and SMA clip displacement relative to their respective mean positions in three vector directions. Left/right (A), anterior/posterior (B), and superior/inferior (C). SMA: Superior mesenteric artery.

**Figure 6:**
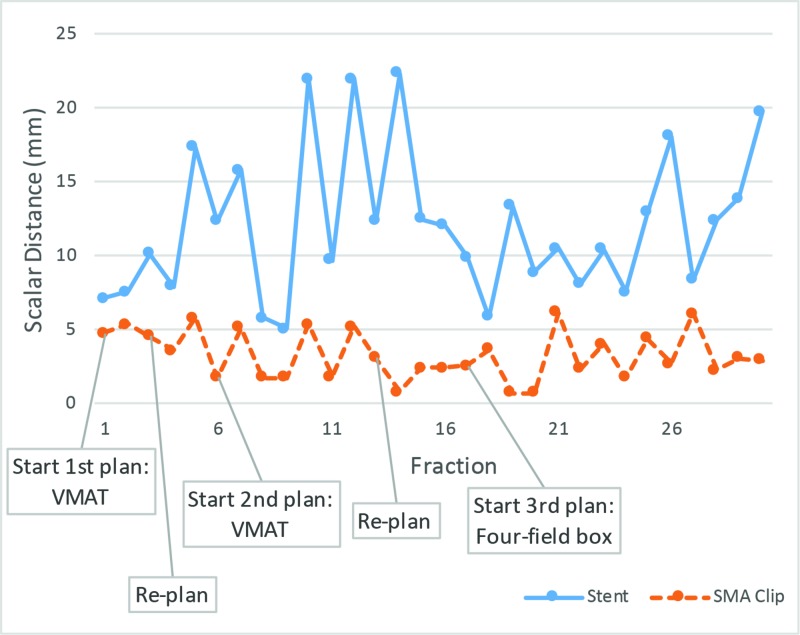
Stent and SMA clip scalar distance relative to their respective mean positions. SMA: Superior mesenteric artery; VMAT: Volumetric modulated arc therapy.

Organ-at-risk (OAR) doses were increased (Figure 4) with the FFB plan, but the entire CTV remained within the high dose region for the remaining fractions. All planning objectives and OAR constraints were met including PTV D99% >= 95%, PTV D0 cc < 105%, liver mean < 30 Gy, liver V30 Gy < 60%, bilateral kidneys mean < 18 Gy, bilateral kidneys V22.5 Gy < 33%, duodenum D0.5 cc < 100%, and spinal canal D0cc < 45 Gy. The mid-stent position on daily CBCT was measured relative to the stable L2 vertebral spinous process. The pancreatic head mass location, inferred by the mid-stent position, varied substantially compared to the SMA node, inferred by the adjacent surgical clip, over all fractions (Figure 5 and Figure 6).

The patient developed grade 2 thrombocytopenia and neutropenia, and gemcitabine was held for the second half of treatment. She also developed grade 2 fatigue, anorexia, and diarrhea which resolved before the completion of treatment. She remained clinically well six months after treatment, with no progression on follow-up CT.

## Discussion

We report a case of locally advanced pancreatic cancer originally planned to be treated with consolidative chemoradiation therapy with VMAT technique. SBRT was avoided due to duodenal invasion on presentation [5]. However, due to substantial inter-fractional motion, the patient was eventually treated with an FFB technique, albeit with higher doses to adjacent organs at risk.

Compared to FFB, conformal techniques have resulted in decreased toxicity, however, they are more sensitive to geometric uncertainties [6,7]. Although the biliary stent is superior to bony anatomy, it is not a perfect surrogate for the GTV. Differences greater than 5 mm between motion of GTV and stent have been observed and therefore a stent should not be relied on as the sole surrogate in highly conformal plans [8,9]. Although metal seed fiducial markers around the pancreatic tumour can help localize the primary GTV, it is an invasive procedure, and does not help localize nodal CTVs, which may move discordantly compared to the primary CTV [10]. Even if the pancreas CTV may be localized, this case shows that shifting to cover pancreas CTV would result in under coverage of the SMA node.

Studies suggest improved overall survival after treating pancreatic cancer with biologically effective doses above 70 Gy [11]. However, inter-fraction motion to the degree observed in this case makes it difficult to deliver higher doses safely unless online daily replanning is used to ensure proper coverage of both the primary and nodal CTV while avoiding critical OARs such as the duodenum. Changes in abdominal anatomy can be detected with daily CT or MR. Preliminary data show excellent outcomes with dose escalation and MRI-guided radiation therapy. Fast daily online adaptive workflows have been developed and daily replanning should account for discordant changes in primary and nodal CTVs and regional OARs, as observed in this case [12-14]. The potential for MRI guidance to improve clinical results is high but requires validation in larger comparative studies.

## Conclusions

In this case report, we presented a woman with a locally advanced pancreatic cancer with a suspicious SMA node and duodenal invasion. Due to her clinical features, SBRT was not an option and therefore we attempted to treat with a conformal VMAT plan. However, because of extensive unpredictable inter-fraction motion, with greater movement at the pancreatic head mass relative to the stable proximal SMA node, the patient was replanned and treated with a four-field box technique. This case highlights the difficulty in delivering conformal radiation while maintaining adequate doses to both the regional nodes and pancreatic head.
